# The potential blunting effect of metformin and/or statin therapy on physical activity‐induced associations with HbA1c in type 2 diabetes

**DOI:** 10.1111/1753-0407.13495

**Published:** 2023-11-14

**Authors:** Joseph Henson, Melanie J. Davies, Emer M. Brady, Charlotte L. Edwardson, Andrew P. Hall, Kamlesh Khunti, Emma Redman, Alex V. Rowlands, Jack Sargeant, Thomas Yates

**Affiliations:** ^1^ NIHR Leicester Biomedical Research Centre Leicester UK; ^2^ Diabetes Research Centre College of Life Sciences, University of Leicester Leicester UK; ^3^ Department of Cardiovascular Sciences University of Leicester Leicester UK; ^4^ Hanning Sleep Laboratory Leicester General Hospital Leicester UK; ^5^ NIHR Applied Health Research Collaboration – East Midlands (NIHR ARC‐EM), Leicester Diabetes Centre Leicester UK; ^6^ Leicester Diabetes Centre University Hospitals of Leicester NHS Trust Leicester UK; ^7^ Alliance for Research in Exercise, Nutrition and Activity (ARENA), UniSA Allied Health and Human Performance University of South Australia, Adelaide, Australia Adelaide South Australia Australia

**Keywords:** metformin, physical activity, statins, type 2 diabetes

## Abstract

**Highlights**

- Our analysis indicates a potential blunting effect of metformin and/or statin therapy on physical activity‐induced associations with HbA1c.
- The benefit of daily physical activity on glycemic control in people with type 2 diabetes is potentially more apparent in those prescribed neither metformin nor statin therapy.
- As physical activity is rarely prescribed in isolation of other background medications used to manage type 2 diabetes, the results of this analysis may help to maximize interventions delivered through routine clinical care, while allowing for personalization in prescribed physical activity and pharmacotherapy.

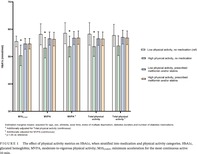

Lifestyle modifications (such as physical activity) are fundamental therapeutic interventions in the management of people living with type 2 diabetes mellitus (T2DM). Similarly, metformin is the established first‐line and thus widely prescribed therapy for T2DM. Their combination would appear complimentary, as they primarily target different tissues (skeletal muscle and liver, respectively). However, experimental data from structured exercise programs suggest that antagonistic interactions may exist between the therapies.^1^, ^2^, ^3^ Likewise, statins, prescribed as standard of care for individuals who have experienced a cardiovascular event and those with type 2 diabetes, may also blunt the adaptations expected with structured exercise.^4^ As previous investigations have typically been conducted in acute, experimental contexts, potential interactions may not necessarily be generalizable to levels of habitual daily activity or reflected in long‐term glycemic control.

## METHODS

As part of the ongoing Chronotype of Patients with Type 2 Diabetes and Effect on Glycaemic Control (CODEC) study 824 participants with established type 2 diabetes (aged 18–75 years) had data collected between 2016 and 2021.^5^ All participants were recruited from primary and specialist health care settings in Leicester, Nottingham, Derby, and Lincoln, United Kingdom. Briefly, participants had established T2DM for more than 6 months, glycated hemoglobin (HbA1c) ≤86 mmol/mol (10%), and were aged between 18 and 75 years.^5^ Ethical approval was obtained from the West Midlands‐ Black Country Research Ethics Committee (16/WM/0457) and all participants gave informed written consent.

### Physical activity

Participants were asked to wear an accelerometer (GENEActiv, ActivInsights Ltd, Kimbolton, UK) on their nondominant wrist, 24 h per day for up to 7 days to quantify physical behaviors. The files were processed using R‐package GGIR version 1.8–1 (http://cran.r-project.org).^6^

Outcomes, averaged across valid days, included time accumulated in sedentary behavior (defined as time accumulated during the waking day below 40 mg), light activity (defined as time accumulated with an acceleration between 40 and 100 mg), moderate‐to‐vigorous physical activity (MVPA) in 1‐min bouts (defined as time accumulated in 1‐min bouts above an acceleration of 100 mg), average acceleration in m*g* (a proxy for total physical activity), and the average acceleration of the most active continuous 10‐min bouts of physical activity (M10_CONT_).

### Statistical analyses

Generalized linear models assessed whether physical activity metrics were associated with HbA1c across medication categories. All models included, as independent variables, age, sex, ethnicity, deprivation, duration of T2DM, number of days the accelerometer was worn, and number of diabetes medications (not including metformin). In the first instance, interaction terms were entered simultaneously into the same model to investigate whether the effect of physical activity metrics (measured continuously) on HbA1c was modified by medication category (prescribed metformin and/or statins, not prescribed). In order to aid interpretability, significant interactions were then stratified into the following groups using median splits: 1 = low physical activity, no prescribed medication (reference) 2 = high physical activity, no prescribed medication, 3 = low physical activity, statins and/or metformin prescribed, 4 = high physical activity, statins and/or metformin prescribed) and presented as estimated marginal means. We also mutually adjusted intensity and volume metrics (MVPA and total physical activity, respectively) for one another. Given the divergent mechanisms of action between metformin and statins, we also ran a sensitivity analysis to examine the associations between physical behavior metrics and HbA1c when stratified into the following groups: prescribed neither metformin or statin therapy; prescribed metformin, not statins; prescribed statins, not metformin; and prescribed both statins and metformin. All data were analyzed using SPSS (version 26.0). *p* values of <.05 and <0.1 (for interactions) were considered statistically significant.

## RESULTS

There were 824 participants with demographic, medication, and device‐measured physical behavior data (mean ± SD or median (interquartile range), age = 63.9 ± 8.3 years, HbA1c = 55 ± 12 mmol/L (7.2 ± 1.2%), sedentary time = 735 ± 102.5 min, and MVPA = 14.9(24.0) min (Table 1).

**TABLE 1:** Participant characteristics.

	All *N* = 824	Not prescribed metformin or statins	Prescribed metformin and/or statins	Prescribed metformin, not statins	Prescribed statins, not metformin	Prescribed metformin and statins
		*N* = 96	*N* = 728	*N* = 146	*N* = 153	*N* = 429
Age (years)	63.9 ± 8.3	63.7 ± 8.8	63.9 ± 8.3	62.0 ± 9.9	64.9 ± 7.9	64.6 ± 7.9
Sex (female)	290 (35.2)	40 (41.7)	250 (34.3)	66 (45.2)	53 (34.6)	131 (30.5)
Ethnicity (white European)	699 (84.8)	79 (82.3)	620 (82.3)	120 (82.2)	131 (85.6)	369 (86.0)
Index of multiple deprivation rank	19 171 ± 9315	20 830 ± 8384	19 108 ± 9363	18 702 ± 9801	17 836 ± 9122	19 687 ± 9263
Current smokers	40 (4.8)	3 (3.1)	37 (5.1)	6 (4.1)	11 (7.2)	20 (4.7)
*Medication*						
Insulin	185 (22.5)	19 (19.8)	166 (22.8)	28 (19.2)	33 (21.6)	105 (24.5)
Metformin	575 (69.8)	‐	575 (79.0)	146 (100)	‐	429 (100)
SGLT2i	74 (9.0)	3 (3.1)	71 (9.8)	12 (8.2)	13 (8.5)	46 (10.7)
DPP‐4i	117 (14.2)	12 (12.5)	105 (14.4)	18 (12.3)	25 (16.3)	62 (14.5)
GLP‐1RA	47 (5.7)	3 (3.1)	44 (6.0)	4 (2.7)	8 (5.2)	32 (7.5)
Sulfonylureas	156 (19.0)	6 (6.3)	150 (20.6)	23 (15.8)	16 (10.5)	111 (25.9)
Statins	582 (70.6)	‐	582 (79.9)	‐	153 (100)	429 (74.6)
Duration of diabetes (years)	10.6 ± 7.5	9.0 ± 7.1	10.8 ± 7.5	9.6 ± 7.8	9.7 ± 7.7	11.6 ± 7.3
BMI (kg/m^2^)	30.9 ± 5.1	30.6 ± 4.9	31.0 ± 5.2	30.7 ± 5.3	30.6 ± 4.9	31.3 ± 5.2
*Cardiometabolic variables*						
HbA1c (mmol/mol)	55 ± 12	53 ± 12	56 ± 12	55 ± 12	53 ± 11	56 ± 12
HbA1c (%)	7.2 ± 1.1	6.9 ± 1.3	7.2 ± 1.1	7.2 ± 1.1	7.0 ± 0.9	7.3 ± 1.1
Total cholesterol (mmol/L)	4.2 ± 1.0	5.0 ± 1.0	4.1 ± 1.0	4.8 ± 1.1	4.2 ± 1.0	3.9 ± 0.9
LDL cholesterol (mmol/L)	2.2 ± 0.8	2.9 ± 0.9	2.1 ± 0.7	2.6 ± 0.8	2.2 ± 0.7	1.9 ± 0.6
*Device‐measured variables*						
Number of valid days	6.9 ± 0.4	6.9 ± 0.4	6.9 ± 0.4	6.9 ± 0.4	6.9 ± 0.4	6.9 ± 0.4
Sedentary time (min)	735.3 ± 102.5	721.0 ± 104.8	737.1 ± 102.1	731.0 ± 97.0	730.0 ± 103.4	741.0 ± 103.4
Light activity (min)	172.4 ± 51.8	180.5 ± 49.8	171.6 ± 51.9	179.8 ± 48.8	162.7 ± 49.9	171.7 ± 53.3
MVPA (>1‐min bouts)	14.9 (24.0)	17.3 (22.4)	14.2 (24.3)	15.1 (27.7)	18.9 (28.1)	12.9 (26.4)
Total physical activity (mg)	21.9 ± 7.0	22.7 ± 5.9	21.8 ± 7.1	22.8 ± 6.7	21.6 ± 7.7	21.5 ± 6.9
M10 _CONT_ (m*g*)	187.9 ± 70.3	188.9 ± 67.1	187.8 ± 70.7	195.7 ± 71.6	193.6 ± 81.4	183.0 ± 65.9

*Note*: Data presented as mean ± SD, median (interquartile range) or number [column percentage].

Abbreviations: BMI, body mass index; DPP‐4i, dipeptidyl peptidase‐4 inhibitor; GLP‐1RA, glucagon‐like peptide‐1 receptor agonist; HbA1c, glycated hemoglobin; MVPA, moderate‐to‐vigorous physical activity; M10_CONT_, minimum acceleration for the most continuous active 10 min; SGLT2i, sodium‐glucose transport protein 2 inhibitor.

**FIGURE 1:**
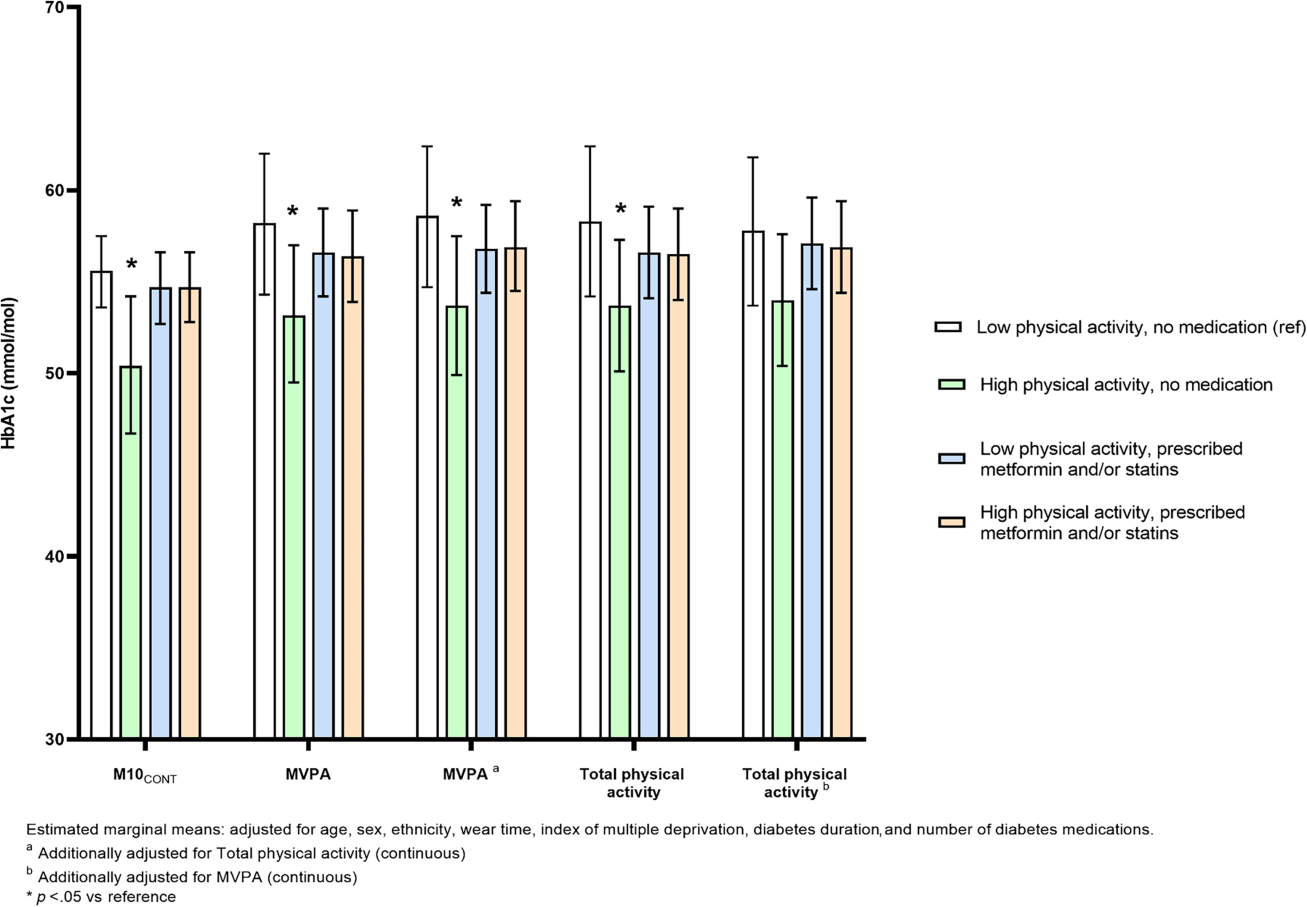
The effect of physical activity metrics on HbA1c, when stratified into medication and physical activity categories. HbA1c, glycated hemoglobin; MVPA, moderate‐to‐vigorous physical activity; M10_CONT_, minimum acceleration for the most continuous active 10 min.

Significant interactions were observed for MVPA (*p* = .020), total physical activity (*p* = .069), and M10_CONT_ (*p* = .052) with HbA1c (measured continuously) but not for light activity or sedentary time. Figure 1 shows the results of the stratified analysis, presented using estimated marginal means. For MVPA, HbA1c was 5.2 mmol/mol (95% confidence interval [CI]: −9.6 to −0.4) lower in the high physical activity, no medication group compared to the reference group (low physical activity, no medication group). Similar results were seen for total physical activity (−4.8 mmol/mol (95% CI: −9.4 to −0.4)) and M10_CONT_ (−3.7 mmol/mol (95% CI: −7.3 to −0.1)). There were no other between‐group differences.

After mutual adjustment for physical activity intensity (MVPA) and volume (total physical activity), the overall pattern was unaffected for intensity. Conversely, the association between volume and HbA1c was attenuated after adjustment for intensity (Figure 1).

**FIGURE 2:**
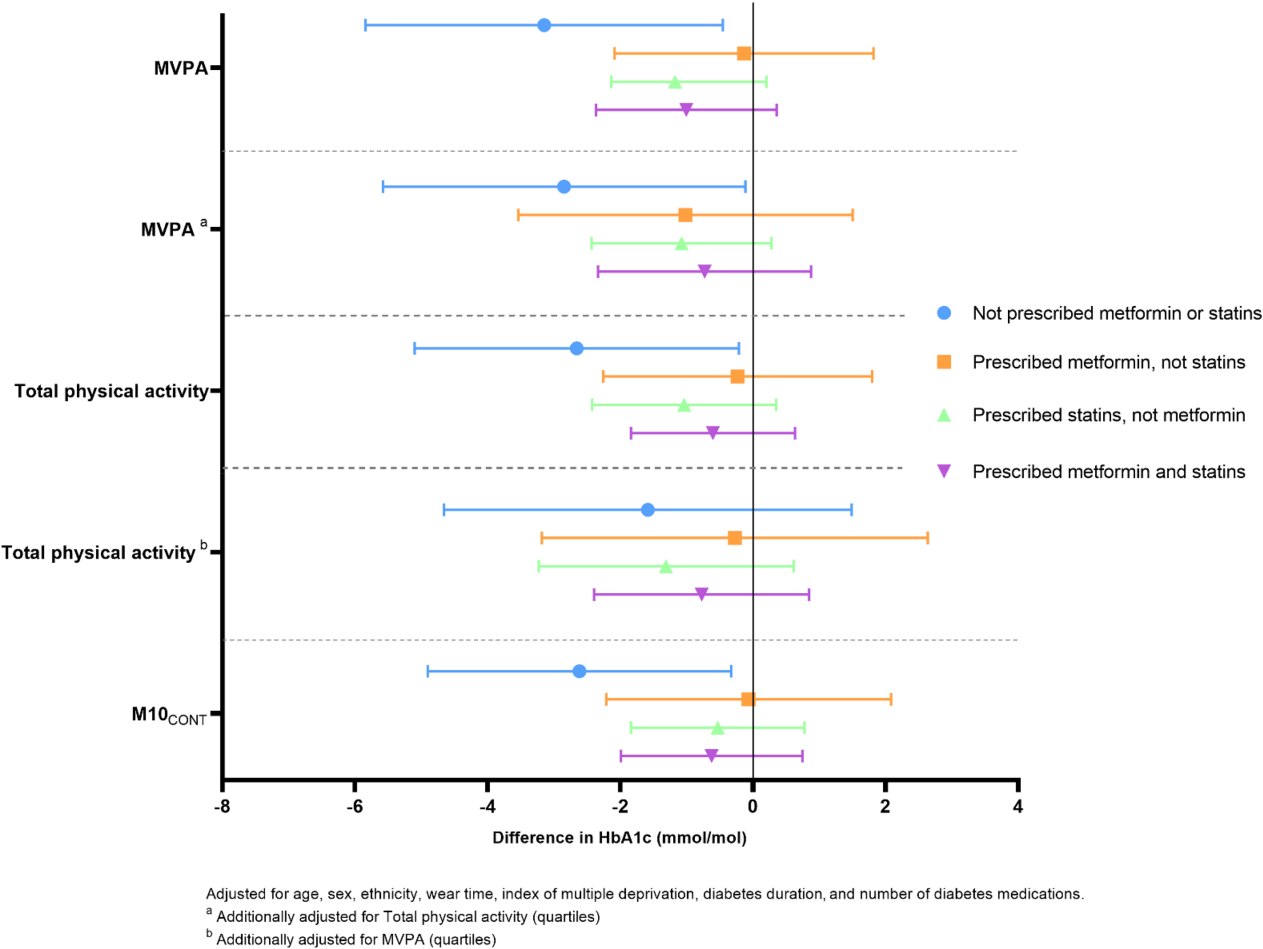
Associations between device measured physical activity metrics (per SD) and HbA1c. HbA1c, glycated hemoglobin; MVPA, moderate‐to‐vigorous physical activity; M10_CONT_, minimum acceleration for the most continuous active 10 min.

The results of the sensitivity analysis demonstrated that the associations between physical activity metrics (per SD) and HbA1c (presented in Figure 2 as differences) were consistent across specific medication categories (prescribed metformin, prescribed statins, prescribed both).

## COMMENT

Our analysis indicates a potential blunting effect of metformin and/or statin therapy on physical activity‐induced associations with HbA1c. Only those individuals in the higher physical activity category, who were not taking medication, displayed lower HbA1c levels compared to the low physical activity, no medication group. Importantly, these findings were independent of key confounders (eg, duration of T2DM, number of other T2DM medications and deprivation). Moreover, total physical activity volume was not associated with HbA1c after adjustment for MVPA, further highlighting the potential importance of movement intensity (vs volume).

Despite the increased interest around the potential interaction between metformin, statins, and physical activity/exercise, the limited evidence is conflicting.^7^, ^8^ Metformin has previously been shown to attenuate exercise‐induced improvements in cardiometabolic risk markers and peripheral insulin sensitivity,^2^, ^3^, ^9^, ^10^ potentially driven by a juxtaposition of exercise and metformin, at the level of the mitochondria. For example, a typical physiological response to meet the energy demands of exercise involves increasing biogenesis and mitochondrial respiration at the site of skeletal muscle. However, when combined with metformin treatment the outcomes are less pronounced (albeit with significant heterogeneity), which may be driven by the inhibition of mitochondrial respiration.^11^ Similarly, statins have been shown to prevent the exercise‐induced increase in mitochondrial content in skeletal muscle.^12^ Our study adds to this emerging body of evidence by suggesting that statin and/or metformin therapy could also blunt the associations between habitual physical activity levels and HbA1c, as an extension to the evidence for exercise training.

The cross‐sectional design limits inference about the direction of causality; as such, reverse causality remains a possibility. We have also analyzed these data as if all medication is the same irrespective of dose and did not directly measure medication compliance. Moreover, wrist‐worn accelerometers may lead to an underestimation of physical activity, particularly when lower‐body movement occurs without simultaneous upper‐body movement (eg, cycling).

Although we included a subgroup of individuals who were not prescribed metformin and/or statins, physical activity is rarely prescribed in isolation of other background medications used to manage T2DM. Therefore, the results of this analysis need to be replicated and investigated further in order to help guide clinical practice.

## DISCLOSURE

The authors report no conflicts of interest.

## Data Availability

The datasets generated during and/or analyzed during the current study are available from the corresponding author on reasonable request.
